# Supplementary figures and images for: Machine learning-assisted elucidation of CD81–CD44 interactions in promoting cancer stemness and extracellular vesicle integrity
Source: eLife. 2022 Oct 4;11:e82669. doi: 10.7554/eLife.82669 (PMC9581534; doi:10.7554/eLife.82669)

Figure 1-source data **1**  
 Uncropped blots associated with Figure 1F

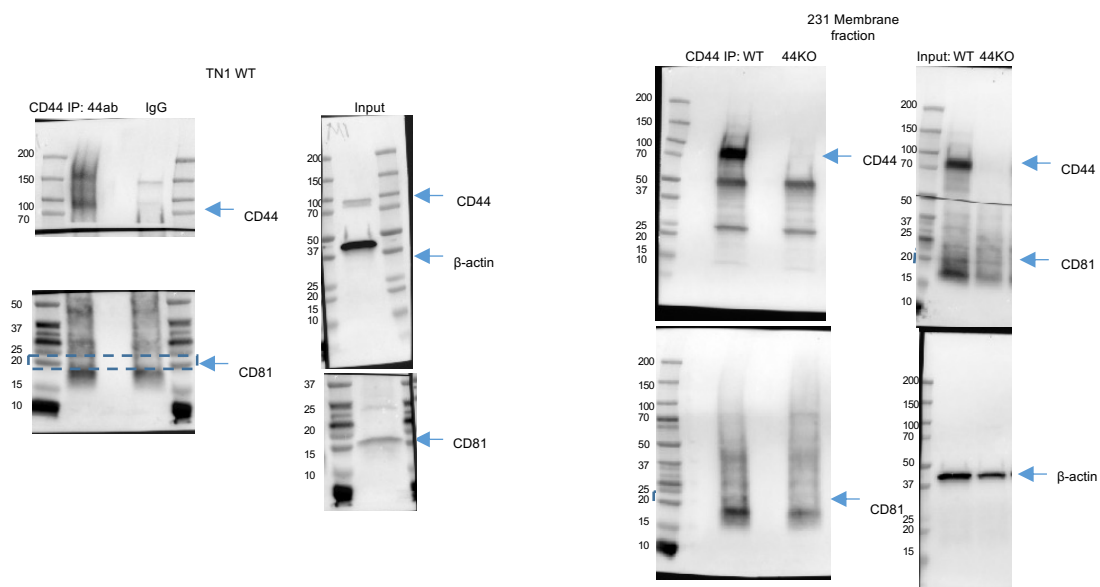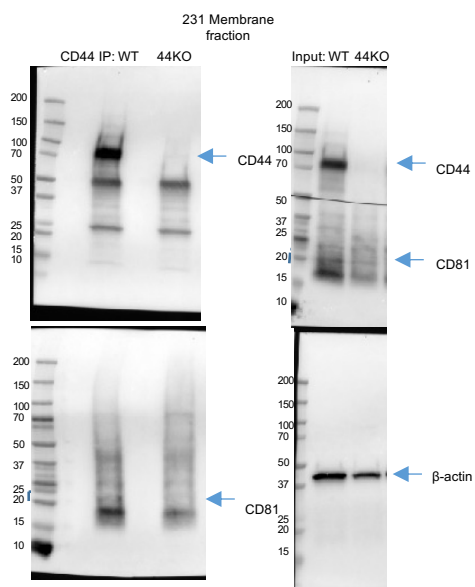

Supplement: Figure 1—source data 1. [file elife-82669-fig1-data1.pdf]

Figure 1-source data 2  
 Uncropped blots associated with Figure 1H

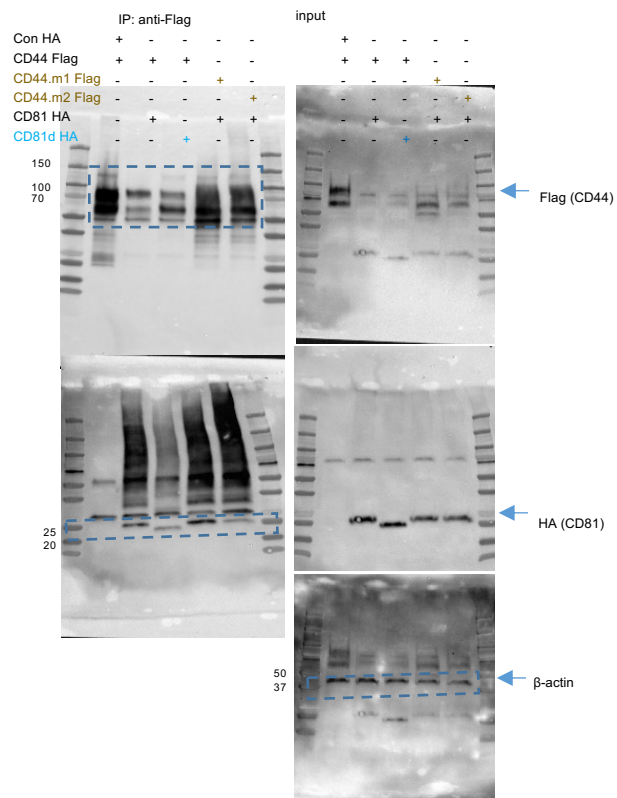

Supplement: Figure 1—source data 2. [file elife-82669-fig1-data2.pdf]

Figure 3-source data 1  
Uncropped blots associated with Figure 3E

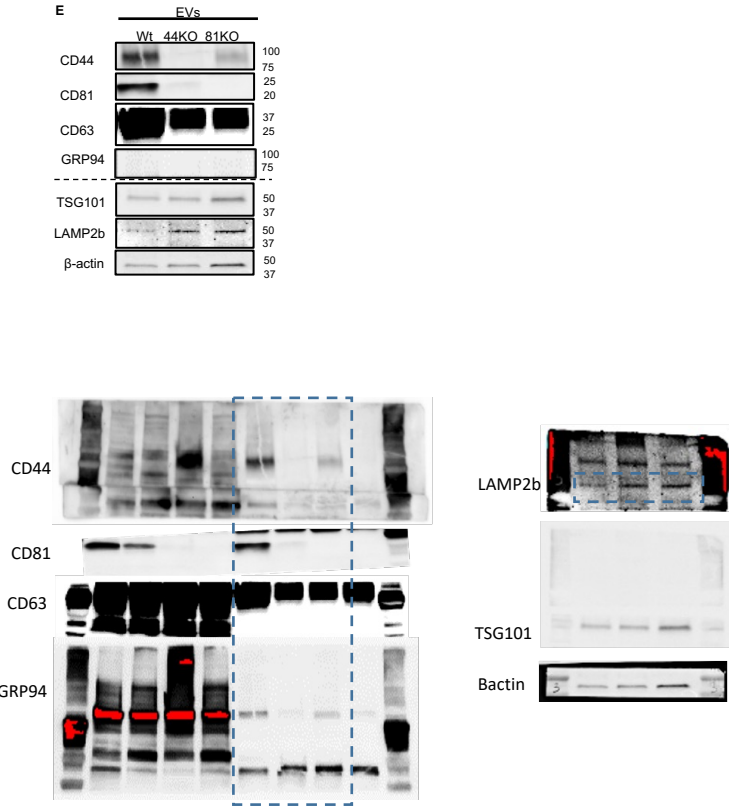

Supplement: Figure 3—source data 1. [file elife-82669-fig3-data1.pdf]

Uncropped blots associated with Figure 3-figure supplement 3

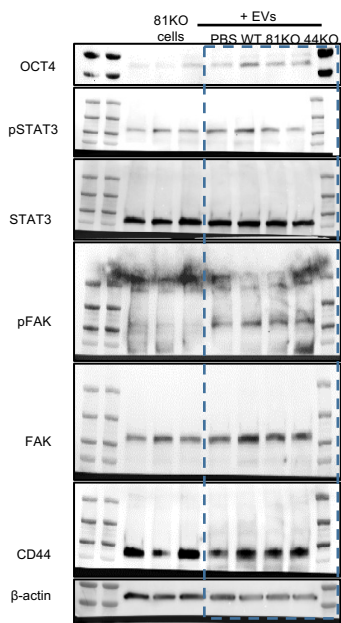

Supplement: Figure 3—figure supplement 3—source data 1. [file elife-82669-fig3-figsupp3-data1.pdf]
