# Supplementary material for: Machine learning-assisted elucidation of CD81–CD44 interactions in promoting cancer stemness and extracellular vesicle integrity
Source: eLife. 2022 Oct 4;11:e82669. doi: 10.7554/eLife.82669 (PMC9581534; doi:10.7554/eLife.82669)

Figure 2-figure supplement 1-source data 2
Uncropped blots associated with Figure 2-figure supplement 1F

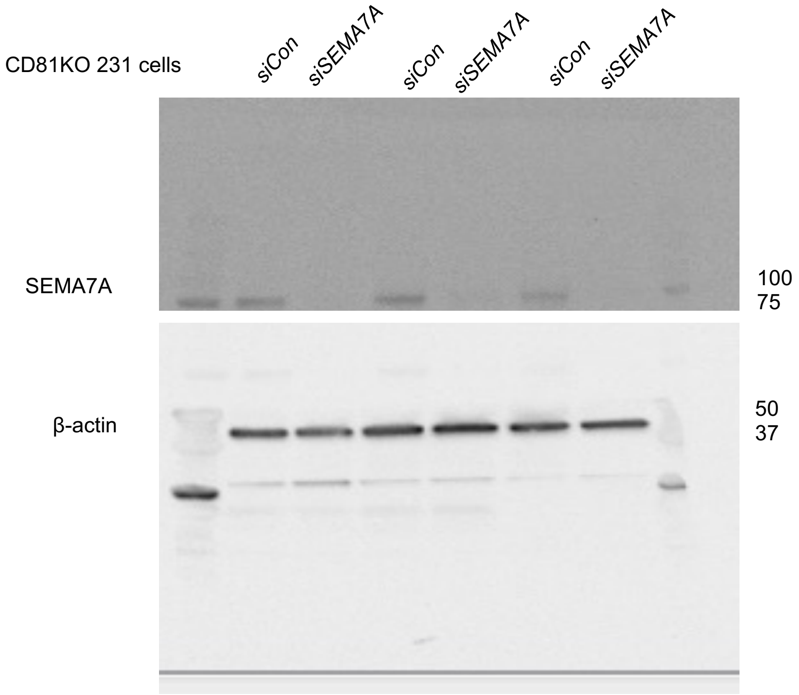

Supplement: Figure 2—figure supplement 1—source data 2. [file elife-82669-fig2-figsupp1-data2.pdf]
